# Questionnaire Breakoff and Item Nonresponse in Web-Based Questionnaires: Multilevel Analysis of Person-Level and Item Design Factors in a Birth Cohort

**DOI:** 10.2196/11046

**Published:** 2018-12-07

**Authors:** Cauane Blumenberg, Daniela Zugna, Maja Popovic, Costanza Pizzi, Aluisio J D Barros, Lorenzo Richiardi

**Affiliations:** 1 Center for Epidemiological Research Post-Graduate Program in Epidemiology Federal University of Pelotas Pelotas Brazil; 2 Cancer Epidemiology Unit Department of Medical Sciences University of Turin Turin Italy

**Keywords:** epidemiology, internet, surveys and questionnaires, epidemiologic research design, data collection

## Abstract

**Background:**

Web-based questionnaires are increasingly used in epidemiologic studies, as traditional methods are facing a decrease in response rates and an increase in costs. However, few studies have investigated factors related to the level of completion of internet-based epidemiologic questionnaires.

**Objective:**

Our objective was to identify person-level characteristics and item design factors associated with breakoff (not finishing the questionnaire) and item nonresponse in a Web-based questionnaire.

**Methods:**

This study was a cross-sectional analysis of the baseline questionnaire, applied from 2005 to 2016, of the Italian NINFEA (Nascita e Infanzia: gli Effetti dell’Ambiente) birth cohort. The baseline questionnaire was administered to enrolled women, who could register at any time during pregnancy. We used logistic regression to analyze the influence of person-level factors on questionnaire breakoff, and a logistic multilevel model (first level: items of the questionnaire; second level: sections of the questionnaire; third level: study participants) to analyze the influence of person-level and item design factors on item nonresponse. Since the number of applicable items depended on the respondent’s characteristics and breakoff, we used inverse probability weighting to deal with missing by design.

**Results:**

Of 5970 women, 519 (8.69%) did not finish the questionnaire. Older age (adjusted odds ratio 1.40, 95% CI 1.05-1.88), lower educational level (adjusted odds ratio [OR] 1.53, 95% CI 1.23-1.90), and earlier stage of pregnancy (adjusted OR 3.01, 95% CI 2.31-3.92) were positively associated with questionnaire breakoff. Of the 1,062,519 applicable items displayed for the participants, 22,831 were not responded to (overall prevalence of item nonresponse 2.15%). Item nonresponse was positively associated with older age (adjusted OR 1.25, 95% CI 1.14-1.38), being in the first trimester of pregnancy (adjusted OR 1.18, 95% CI 1.06-1.31), and lower educational level (adjusted OR 1.23, 95% CI 1.14-1.33). Dropdown menu items (adjusted OR 1.77, 95% CI 1.56-2.00) and items organized in grids (adjusted OR 1.69, 95% CI 1.49-1.91) were positively associated with item nonresponse.

**Conclusions:**

It is important to use targeted strategies to keep participants motivated to respond. Item nonresponse in internet-based questionnaires is affected by person-level and item design factors. Some item types should be limited to reduce item nonresponse.

## Introduction

### Background

Novel data collection methods are increasingly used in epidemiologic studies [1,2], as traditional methods, including mail questionnaires, face-to-face interviews, and telephone interviews, are facing a decrease in response rates [3] and an increase in costs [4]. Given the limitations of traditional methods and the growing internet penetration, the number of Web-based e-epidemiologic studies is increasing worldwide [1].

Compared with traditional methods, Web-based epidemiologic questionnaires have clear advantages, such as higher data quality (if filtering questions and consistency checks are used) and lower costs [1]. However, they may also have weaknesses that should be explored empirically [5]. In particular, the validity of epidemiologic studies may be jeopardized by lower response rates [6], questionnaire breakoff (not finishing the questionnaire), and item nonresponse [7], which can depend on participants’ characteristics and item design factors [8,9]. For instance, in a study investigating homosexual rights, the item nonresponse rates were higher among heterosexual individuals than among homosexual individuals [10]. In this case, the item nonresponse rates varied according to individual characteristics that were relevant to the objectives of the study, and this could bias the results [11].

### Objective

Although item nonresponse may have a great impact on study validity, few studies have investigated factors related to the level of completion of internet-based epidemiologic questionnaires [12,13]. Thus, in the context of the internet-based NINFEA (Nascita e Infanzia: gli Effetti dell’Ambiente) birth cohort study [14], we aimed at investigating the associations of person-level characteristics and item design factors with item nonresponse rate, as well as the associations of person-level characteristics with questionnaire breakoff.

## Methods

### Participants and Baseline Questionnaire

NINFEA is a Web-based birth cohort study, which started in Italy in 2005 [14]. Members of the cohort are children born to women who (1) had enough knowledge of the Italian language to complete internet-based questionnaires, (2) knew about the study, and (3) had access to the internet at the time of recruitment. Participants were recruited online through hyperlinks leading to the NINFEA website that were displayed on selected hospitals’ home pages, pregnancy-related websites, and the NINFEA Facebook page, and offline using leaflets, face-to-face contacts, and posters placed in selected hospitals and clinics. The study was also advertised in local and national media in Italy. All selected hospitals and clinics for online and offline recruitment were located in the Piedmont and Tuscany regions, from which 82.87% (6391/7712) of the cohort originated. Pregnant women could enroll by registering at the study website [15] at any time during pregnancy. The ethical committees of the San Giovanni Battista Hospital and the Orthopedic Traumatology Center, Functional Re-education Center, Maria Adelaide Hospital, Turin, Italy (approval #0048362 and following amendments) approved the study, and all participants consented to participate. At enrollment, they completed a baseline questionnaire, and then were invited to fill in 5 follow-up questionnaires when their child turned 6 months, 18 months, 4 years, 7 years, and 10 years of age. This study focused on the baseline questionnaire.

In the period from 2005 to 2016, a total of 7712 pregnant women completed the NINFEA baseline questionnaire (database version 03.2017), and 1176 women participated during more than 1 pregnancy. The questionnaire was initially developed using the Hypertext Preprocessor scripting language [16]. After the first 1500 respondents, a major review of the questions was done and an updated version of the questionnaire was implemented using the Ruby programming language [17]. To avoid comparability issues, for this study we considered only the 5970 pregnant women who completed at least one section of the Ruby version.

The baseline questionnaire is composed of 18 sections investigating demographic factors, maternal general health, exposures before and during pregnancy, lifestyle, and reproductive history. Of these sections, 4 are supplementary and entirely dependent on answers given in the preceding section, and thus we did not consider them in the analyses. In total we included 244 items in the 14 analyzed sections; of these, 7 items were mandatory and therefore we excluded them from the analyses. We thus analyzed a total of 237 items, although the actual number of items presented to each participant at the time they completed the questionnaire varied due to filter questions that render sets of questions not applicable. For example, a negative answer to the filter question “Did you smoke during pregnancy?” would skip a series of questions about smoking. In contrast, a positive answer to the same filter question would present a set of applicable questions about smoking to the respondent.

### Questionnaire Breakoff and Item Nonresponse

We analyzed 2 outcomes: questionnaire breakoff and item nonresponse. We considered a respondent to have broken off the questionnaire if she stopped answering the items before reaching the last section. If the last section was fully or partially completed and submitted, we considered the questionnaire not to be broken off, even if some items were left blank in the preceding sections. For this reason, no breakoff could have occurred in the last section of the questionnaire. For the analysis of questionnaire breakoff, the units of analysis were the 5970 women who completed at least one section of the questionnaire.

We based the analyses of item nonresponse on the 237 nonmandatory items from the 14 sections of the questionnaire. We assessed each of the 237 nonmandatory items, for each of the 5970 participants, and considered a blank as a nonresponse if the item was applicable. Item nonresponse was constructed as a binary variable: 1=nonresponse, and 0=response. The units of analysis were the items of the questionnaire (at most 237 items × 5970 women = 1,414,890 items).

We analyzed the following person-level characteristics as predictors of questionnaire breakoff: age (≤30 years, 31-35 years, ≥36 years), university degree (yes, no), gestational trimester at enrollment (first trimester, second trimester, third trimester), first pregnancy (yes, no), employment status at the beginning of the pregnancy (employed, unemployed), type of recruitment (offline, online), Italian region of residence (Piedmont Region, Tuscany Region, other regions of Northern Italy, and other), and number of participations in the baseline questionnaire (1, ≥2). All the exposure variables were self-reported in the baseline questionnaire, except for the number of participations, which was constructed based on the total number of baseline questionnaires compiled by a woman. We assessed the type of recruitment from the first question, which asked about the way the participant had become aware of the study. We considered leaflets, posters, word-of-mouth, face-to-face invitation, and traditional media as offline recruitment methods, while we considered built-in links in websites and social media sites as online recruitment methods. Specifically, for the online recruitment, we advertised the study in selected forums or websites targeting pregnant women or health care workers, on the home pages of selected obstetric or pediatric hospitals or hospitals with a large number of deliveries, and on the NINFEA Facebook page. The number of involved websites, forums, and hospitals changed over time depending on the specific type of collaboration that was initiated. We conducted two small Facebook campaigns with advertisements targeting women in fertile age [18].

We assessed item nonresponse in association with the person-level characteristics analyzed for questionnaire breakoff, as well as in association with the design of the items themselves: (1) item type (checkbox, dropdown menu, radio button, text), (2) number of response options, and (3) whether the item was located in a grid (yes, no). Multimedia Appendix 1 provides examples of the item design characteristics. Specifically, radio button items can have only 1 answer selected among a set of predefined response options; dropdown menu items also have only 1 possible answer, but the list of response options is collapsed by default and has to be actively expanded to read the possible responses; checkboxes accept the selection of more than 1 answer from a set of predefined response options; and text items require the insertion of numeric or textual content. Some items in the questionnaire combined a radio button or a checkbox with a text item (eg, items with response options “Other, namely...”); these were considered as 2 individual items. We categorized the number of response options as 2, 3 to 5, and at least 6 options; we did not consider text items because they do not have any response option. An item was considered to be located in a grid if it was part of a group of items that shared the same set of response options and that required the respondents to link rows and columns in order to select an appropriate answer.

### Statistical Analyses

We estimated the odds ratios (ORs) and 95% confidence intervals of breaking off the questionnaire according to person-level factors by using logistic regression with robust variance estimation to account for the correlation between the responses of mother who participated in the NINFEA cohort during more than 1 pregnancy.

To analyze the association of person-level and item design factors with item nonresponse, we used a 3-level hierarchical logistic regression model. The questionnaire items composed the first level, the questionnaire sections were the second level, and the women responding to the questionnaire were the third level. We fitted crude and adjusted models, by adjusting mutually for maternal age, university degree, employment status, gestational trimester, whether it was a first pregnancy, type of recruitment, region of residence, and number of participations.

As filters were used in the questionnaire, the total number of items to be responded to varied among participants. To account for these differences, we applied the inverse probability weighting (IPW) technique to deal with data missing by design [19]. In this study, we calculated the weights as the inverse of the probability of having a missing datum (by design) on every dependent item by considering only the women for whom that item was applicable. We estimated the weights using a logistic regression model that included the following person-level characteristics: age, university degree, gestational trimester at enrollment, whether it was a first pregnancy, employment status at the beginning of the pregnancy, and the type of recruitment. The underlying idea of IPW is to create weighted copies of the complete cases (dependent applicable items), according to selected person-level characteristics, to remove the selection bias introduced by the missing data. By doing so, we assumed that the nonresponse probability of women for whom the item was not applicable was equal to the nonresponse probability of women for whom the item was applicable, given that they had the same selected person-level characteristics. We did not truncate high-weight values, as, in sensitivity analyses, truncation at the 95th or 99th percentile did not affect the results more than marginally.

Analyses were conducted using the Stata 15.0 software (StataCorp LLC).

## Results

### Participant Characteristics

Table 1 lists the main characteristics of the 5970 women included in the analyses. Most of the NINFEA participants lived in the Piedmont Region, were recruited offline, and were in the third trimester of pregnancy. Two-thirds of women were younger than 35 years (n=4235), and more than half had a university degree (n=3605), were employed (n=5067), or were in their first pregnancy (n=3196). A total of 1176 women participated with more than 1 pregnancy in the NINFEA birth cohort.

**Table 1:** Characteristics of the study population (N=5970).

Participant characteristics		n (%)^a^
**Age group (years)**		
	≤30	1735 (29.06)
	31-35	2505 (41.96)
	≥36	1730 (28.98)
**University degree**		
	Yes	3605 (61.59)
	No	2248 (38.41)
**Employment status**		
	Unemployed	903 (15.13)
	Employed	5067 (84.87)
**Gestational trimester**		
	First	968 (16.41)
	Second	1798 (30.48)
	Third	3133 (53.11)
**First pregnancy**		
	Yes	3196 (53.58)
	No	2769 (46.42)
**Type of recruitment**		
	Offline	4839 (83.71)
	Online	942 (16.29)
**Region of residence**		
	Piedmont Region	3328 (56.14)
	Tuscany Region	1720 (29.01)
	Other regions of North Italy	500 (8.43)
	Other	380 (6.41)
**Number of participations**		
	1	4794 (80.30)
	≥2	1176 (19.70)

^a^Total numbers may vary due to missing values.

### Questionnaire Breakoff and Item Nonresponse Characteristics

Table 2 shows the number of sections, item characteristics, and nonresponse percentage according to item design characteristics. We analyzed a total of 237 items from 14 sections in this study. Almost half of the items (n=116) were radio button type and included 3 to 5 response options. Of the 237 items, 39 (16.5%) were located in a grid. The highest nonresponse percentages among the applicable items were observed for filter questions, dropdown menu items, items containing 3 to 5 response options, and items located in grids.

Of the 5970 women, 519 (8.69%) did not finish the NINFEA baseline questionnaire. Breakoffs were spread over the 13 sections of the questionnaire. Table 3 shows the ORs of breakoff depending on the participants’ characteristics. Women who at enrollment were in the first trimester of pregnancy had a threefold higher odds of questionnaire breakoff than did those who were in the third trimester of pregnancy (adjusted OR 3.01, 95% CI 2.31-3.92). Women without a university degree had 53% higher odds of questionnaire breakoff (95% CI 1.23-1.90) than did those with a higher education. Older age was also positively associated with questionnaire breakoff.

**Table 2:** Characteristics of the questionnaire items and frequency of nonresponse according to item characteristics.

Item characteristics		n (%)	Nonresponse, n (%)^a^
Sections		14	N/A^b^
Items		237	22,831 (2.15)
**Filter question**			
	No	148 (62.4)	3900 (1.84)
	Yes	89 (37.6)	18,931 (2.22)
**Item type**			
	Checkbox	14 (5.9)	804 (1.48)
	Dropdown menu	49 (20.7)	7454 (2.84)
	Radio button	116 (48.9)	12,335 (2.17)
	Text (open question)	58 (24.5)	2238 (1.26)
**Number of response options^c^**			
	2	69 (38.6)	7606 (2.20)
	3-5	85 (47.4)	11,827 (2.65)
	≥6	25 (14.0)	1160 (1.27)
**Item in a grid**			
	No	198 (83.5)	16,625 (1.96)
	Yes	39 (16.5)	6206 (2.92)

^a^Calculated as the ratio between the total number of items not responded to and the total number of applicable items (n=1,062,519) for all participants.

^b^N/A: not applicable.

^c^Text items were not considered.

**Table 3:** Questionnaire breakoff according to participants’ characteristics.

Participant characteristics		n (%)	Crude analyses, OR^a^ (95% CI)	Adjusted analyses^b^, OR (95% CI)
**Age group (years)**				
	≤30	137 (7.9)	1.00	1.00
	31-35	213 (8.5)	1.08 (0.87-1.36)	1.11 (0.84-1.44)
	≥36	169 (9.8)	1.26 (1.00-1.60)	1.40 (1.05-1.88)
**University degree**				
	Yes	220 (6.1)	1.00	1.00
	No	206 (9.2)	1.55 (1.27-1.90)	1.53 (1.23-1.90)
**Employment status**				
	Employed	363 (7.2)	1.00	1.00
	Unemployed	156 (17.3)	2.71 (2.21-3.32)	0.99 (0.73-1.34)
**Gestational trimester**				
	Third	189 (6.0)	1.00	1.00
	Second	134 (7.5)	1.25 (1.00-1.58)	1.27 (0.98-1.65)
	First	170 (17.6)	3.32 (2.65-4.15)	3.01 (2.31-3.92)
**First pregnancy**				
	Yes	233 (7.3)	1.00	1.00
	No	286 (10.3)	1.47 (1.22-1.76)	1.13 (0.90-1.43)
**Type of recruitment**				
	Offline	389 (8.0)	1.00	1.00
	Online	107 (11.4)	1.47 (1.17-1.84)	1.11 (0.82-1.51)
**Region of residence**				
	Piedmont Region	236 (7.1)	1.00	1.00
	Tuscany Region	170 (9.9)	1.44 (1.17-1.77)	1.06 (0.84-1.35)
	Other regions of North Italy	49 (9.8)	1.42 (1.03-1.97)	1.14 (0.75-1.73)
	Other	54 (14.2)	2.17 (1.58-2.99)	1.80 (1.21-2.66)
**Number of participations**				
	1	387 (8.1)	1.00	1.00
	≥2	132 (11.2)	1.44 (1.17-1.77)	1.19 (0.91-1.57)

^a^OR: odds ratio.

^b^Models adjusted for age, university degree, employment status, gestational trimester, first pregnancy, type of recruitment, region, and number of participations.

Of the 1,062,519 applicable items, 22,831 were not responded to, giving an overall item nonresponse rate of 2.15%. Table 4 presents the weighted crude and adjusted ORs of item nonresponse according to participants’ characteristics. Similar to the findings for questionnaire breakoff, lower educational level, older age, and enrollment in the first trimester of pregnancy were positively associated with item nonresponse. In contrast, participating during 2 or more pregnancies (ie, responding to the questionnaires twice or more often) was associated with lower odds of item nonresponse. Number of pregnancies, employment status, and type of recruitment were not associated with item nonresponse in our study.

All the analyzed item design factors were associated with item nonresponse (Table 5). Items designed as a dropdown menu were 77% more likely to be left blank than were radio button items (95% CI 1.56-2.00). Text items had 30% lower odds of item nonresponse (95% CI 0.63-0.79) and checkboxes had 80% lower odds of item nonresponse (95% CI 0.16-0.25) than did radio button items. Items with 6 or more response options were 59% less likely to be left blank than were those with 2 response options (95% CI 0.35-0.47). Finally, items being located in a grid was positively associated with nonresponse (adjusted OR 1.69, 95% CI 1.49-1.91).

**Table 4:** Prevalence and crude and adjusted odds ratios (ORs) of item nonresponse according to participants’ characteristics.

Participant characteristics		Prevalence (%)	Crude analyses, OR (95% CI)	Adjusted analyses^a^, OR (95% CI)
**Age group (years)**				
	≤30	2.1	1.00	1.00
	31-35	2.0	1.03 (0.95-1.13)	1.07 (0.98-1.17)
	≥36	2.4	1.25 (1.14-1.38)	1.25 (1.14-1.38)
**University degree**				
	Yes	1.9	1.00	1.00
	No	2.4	1.22 (1.14-1.31)	1.23 (1.14-1.33)
**Employment status**				
	Employed	2.0	1.00	1.00
	Unemployed	3.0	0.89 (0.78-1.01)	0.87 (0.77-0.98)
**Gestational trimester**				
	Third	2.0	1.00	1.00
	Second	2.1	1.04 (0.96-1.12)	1.00 (0.93-1.09)
	First	2.6	1.17 (1.06-1.29)	1.18 (1.06-1.31)
**First pregnancy**				
	Yes	2.2	1.00	1.00
	No	2.1	1.05 (0.98-1.12)	1.03 (0.95-1.11)
**Type of recruitment**				
	Offline	2.1	1.00	1.00
	Online	2.4	1.12 (1.01-1.23)	1.07 (0.96-1.18)
**Region of residence**				
	Piedmont Region	1.9	1.00	1.00
	Tuscany Region	2.5	1.17 (1.08-1.27)	1.16 (1.07-1.25)
	Other regions of North Italy	1.9	1.02 (0.90-1.15)	0.97 (0.85-1.11)
	Other	2.8	1.37 (1.16-1.61)	1.14 (0.98-1.34)
**Number of participations**				
	1	2.2	1.00	1.00
	≥2	1.9	0.84 (0.77-0.92)	0.90 (0.82-0.99)

^a^Models adjusted for age, university degree, employment status, gestational trimester, first pregnancy, type of recruitment, region, and number of participations.

**Table 5:** Crude and adjusted odds ratios (ORs) of item nonresponse according to item design factors.

Item design factors		Crude analyses, OR (95% CI)	Adjusted analyses^a^, OR (95% CI)
**Item type**			
	Radio button	1.00	1.00
	Checkbox	0.20 (0.17-0.25)	0.20 (0.16-0.25)
	Dropdown menu	1.73 (1.53-1.94)	1.77 (1.56-2.00)
	Text (open question)	0.70 (0.63-0.78)	0.70 (0.63-0.79)
**Response options**			
	2	1.00	1.00
	3-5	1.12 (1.04-1.21)	1.09 (1.01-1.18)
	≥6	0.41 (0.35-0.47)	0.41 (0.35-0.47)
**Item in a grid**			
	No	1.00	1.00
	Yes	1.63 (1.44-1.83)	1.69 (1.49-1.91)

^a^Models adjusted for age, university degree, employment status, gestational trimester, first pregnancy, type of recruitment, region, and number of participations.

## Discussion

### Principal Findings

Our results showed that women enrolled in earlier stages of pregnancy had a higher probability of questionnaire breakoff than did women enrolled in the third trimester of pregnancy. Older and less-educated women were more likely to break off the questionnaire and to leave items blank. Dropdown menu items were associated with the lowest response rate among all types of items. Unexpectedly, text items were less likely to be left blank than were radio button items; similarly, items with 6 or more response options were less likely to be left blank than were those with 2 response options.

Our findings of higher breakoff and item nonresponse rates among women in the first trimester of pregnancy than among those enrolled in the third trimester could be explained by several factors, including participants’ time available to answer the questionnaire. Women in later stages of pregnancy might have more time to complete the questionnaire, as they are already on maternity leave. Lower educational level was positively associated with questionnaire breakoff in the NINFEA Web-based cohort. This finding is consistent with other studies that included different populations (eg, men) [20,21] or used different data collection methods, such as postal questionnaires [22]. These consistencies are of particular interest, as the NINFEA study population includes self-selected volunteers having access to the internet; nevertheless, differences in completion of the questionnaire by educational level persist. Thus, regardless of the population or data collection method, epidemiologic studies that rely on self-administered questionnaires should identify incentives to motivate participation, specifically of individuals with low educational levels.

In contrast, there are determinants that are closely related to Web-based studies, such as whether the participants became aware of the study through online or offline channels. Few studies have investigated the associations between the type of recruitment and breakoff from internet-based questionnaires [23]. Our finding of no association is in line with the findings of an internet-based intervention that found no difference in questionnaire breakoff between online and offline recruitment methods [24].

The proportion of item nonresponse was low in our study, ranging from 1.3% to 2.9%. Another study that administered daily Web-based questionnaires also described low rates of item nonresponse, ranging from 0% to 7.4% [25]. In our study, online recruitment, older age, and lower educational levels were positively associated with item nonresponse. This is in line with findings of 3 quality-of-life Web-based surveys conducted in the United States [26]. The association between older age and lower educational levels with higher rates of item nonresponse is also consistent with other prior work [27,28]. Regardless of the data collection method used, these individuals have to expend a higher cognitive effort to respond to questions. In the case of a self-reported questionnaire responded to over the internet (with no support from an interviewer), the rates of nonresponse for these individuals can be even higher.

The number of times a woman participated in the NINFEA baseline questionnaire was not associated with breakoff, but it was associated with lower rates of item nonresponse. However, the confidence interval almost included the unit, and for this reason we believe this association might be due to residual confounding.

To analyze item nonresponse according to the type of item, we compared all items with the radio button items, since this was the most prevalent item in the NINFEA questionnaire. Our finding that checkbox items were associated with a lower item nonresponse than the radio button items is consistent with the literature and inherent in the logic of checkboxes [26,29]. The probability of checking at least 1 answer among several response options is likely higher than checking 1 answer among a pair of response options [29]. Our finding of lower item nonresponse among items with 6 or more response options than among items with 2 response options supports this hypothesis. Text items were associated with a higher response than were radio button items in our study. The association of text items with item nonresponse is still controversial in the literature, as studies found text items to be positively or negatively associated with item nonresponse [26,30]. Dropdown menu items were positively associated with item nonresponse, as they require more actions to select an answer (3 actions for dropdown menu items vs 1 action for radio button items), and this can explain the higher item nonresponse rate [11,31].

As expected, items located in grids had higher odds of item nonresponse than did single items. Linking rows and columns of a grid to select an appropriate answer is more complex than choosing an answer of a single item; hence, if possible, grid items should be avoided [32,33].

Besides the design of the items, their content could also influence item nonresponse [26]. For instance, items asking about sensitive subjects could have higher nonresponse than items with nonsensitive content [34]. However, we did not perceive this behavior in our study. In the NINFEA baseline questionnaire, we considered only 3 of the 237 items to have sensitive content: alcohol consumption during pregnancy, use of soft drugs during pregnancy, and smoking during pregnancy. There were no missing responses for the first 2 items and 9 missing responses for the item asking about smoking.

### Conclusion

We obtained our findings within the context of a longitudinal epidemiologic study: the NINFEA Web-based birth cohort. In this type of study, it is very important to avoid breakoffs and item nonresponse, since the presence of missing values in the baseline questionnaires makes analyses of future outcomes difficult. Using the IPW technique and multilevel modeling, we were able to comprehensively and concurrently analyze the association of person-level and item design factors with item nonresponse. By doing so, we were also able to adjust all analyses for the characteristics of the mothers.

To our knowledge, this is the first study evaluating determinants of questionnaire breakoff and item nonresponse in the context of e-epidemiology. Our study was based on only 1 internet-based epidemiologic study and included only pregnant women; thus, replications in other populations and settings are needed. It is crucial to understand the profile of nonresponders to develop personalized motivation methods and minimize item nonresponse and breakoffs. Personalized recruitment [35,36], use of reminders [37,38], incentives [39,40], and gamification [41] are only some of the strategies that can be used to keep participants motivated.

The low percentage of breakoffs in the baseline questionnaire of the NINFEA birth cohort demonstrates the feasibility of e-epidemiologic research, even when long questionnaires are applied. However, the questionnaires should be designed carefully. For instance, items with 1 and several radio button options should replace dropdown menu items and items located in grids, respectively, in order to reduce nonresponse. Also, we showed several person-level characteristics to be important determinants of breakoff and item nonresponse in internet-based questionnaires. For this reason, study coordinators should know their target population so as to employ focused motivation and recruitment techniques and to reduce breakoff and item nonresponse. Older and less educated individuals should be contacted directly (even by other means, such as telephone) in order to assist and encourage their participation in e-epidemiologic research.

## Appendix

Multimedia Appendix 1 Examples of item characteristics.

## Abbreviations

IPW: inverse probability weighting
NINFEA: Nascita e Infanzia: gli Effetti dell’Ambiente
OR: odds ratio
