# Supplementary material for: Questionnaire Breakoff and Item Nonresponse in Web-Based Questionnaires: Multilevel Analysis of Person-Level and Item Design Factors in a Birth Cohort
Source: J Med Internet Res. 2018 Dec 7;20(12):e11046. doi: 10.2196/11046 (PMC6303736; doi:10.2196/11046)
Supplement: Multimedia Appendix 1 [file jmir_v20i12e11046_app1.pdf]

Multimedia Appendix 1: Examples of the item characteristics: (a) radio item with two response options; (b) checkbox item with three response options; (c) dropdown item with five response options; (d) text item; and (e) three radio items located in a grid with two response options each. Radio and dropdown items can only have one response option selected, while checkbox items can have more than one answer selected.

(a)

During the first 12 months of pregnancy, did you follow any diet to lose weight?

- ○ Yes
- ○ No

(b)

Select which of the following diseases you have been diagnosed by a doctor:

- ☐ Asthma
- ☐ Rhinitis
- ☐ Bronchitis
- ☐ None of the above

(c)

During the first three months of pregnancy, how often you used pesticides?

▼

- Never
- Less than one time per week
- One time per week
- From two to six times per week
- Everyday

(d)

What is your height? [ ]

(e)

During the first three months of pregnancy, did you have contact with someone with…

	**Yes**	**No**
…tuberculosis?	○	○
…varicella?	○	○
…cytomegalovirus?	○	○
